# Invited papers - Clinical Trial Updates Session

**DOI:** 10.1054/bjoc.2000.1347

**Published:** 2000-08-10


*British Journal of Cancer* (2000) **83**(Suppl 1), 1–2
© 2000 Cancer Research Campaign
doi: 10.1054/ bjoc.2000.1347, available online at http://www.idealibrary.com on IDEAL®

### CT1 ESPAC-1 INTERIM RESULTS: A EUROPEAN, RANDOMISED STUDY TO ASSESS THE ROLES OF ADJUVANT CHEMOTHERAPY (5 FU+FOLINIC ACID) AND ADJUVANT CHEMORADIATION (40 GY+5 FU) IN RESECTABLE PANCREATIC CANCER

JP Neoptolemos*[1], JA Dunn[2], DD Moffitt[2], J Almond[1], HG Beger[3], KH Link[3], P Pederzoli[4], C Bassi[4], C Dervernis[5], L Fernandez-Cruz[6], F Lacaine[7], D Spooner[8], DJ Kerr[2], H Freiss[9], MW Büchler[9], [1]Royal Liverpool University Hospital, Daulby St, Liverpool L69 3GA, UK, [2]CRC Institute for Cancer Studies, Birmingham, UK, [3]Ulm University Hospital of Surgery, Germany, [4]University of Verona, Italy, [5]Agia Olga Hospital, Athens, Greece, [6]Barcelona University Hospital, Spain, [7]Hopital Tenon, Paris, France, [8]Birmingham Oncology Centre, UK, [9]University of Berne, Switzerland

Pancreatic cancer affects 8–12 per 100,000 population per year in Europe and North America. Post-resection, long term survival is only 10–15% and the role of adjuvant treatment is uncertain.

Previously GITSG randomised 43 patients showing increased survival in patients receiving chemoradiation (40 Gy+5 FU) then weekly 5 FU compared to controls. The Norwegian Pancreatic Cancer Trial Group randomised 47 patients showing increased median survival in chemotherapy (FAM) patients compared to controls but no overall survival difference. The EORTC trial of 114 patients did not show an advantage for postoperative chemoradiation (40 Gy+5 FU) compared to controls but the UK Pancreatic Cancer Group concluded that the GITSG regimen might enhance survival in patients with negative lymph nodes.

ESPAC-1 is the largest randomised adjuvant pancreatic study aiming to answer two questions: (i) is there a role for chemoradiation (40 Gy+5 FU); (ii) is there a role for chemotherapy (weekly 5 FU/FA). So far, 530 patients with pancreatic ductal adenocarcinoma have been randomised from 80 clinicians in 11 countries. Presently, 239 patients (45%) are alive with median follow-up of 9 (IQR 1–24) months.

Preliminary results show no evidence of a benefit for chemoradiation treatment (median survival 14 months with chemoradiation vs 15.7 months without, p=0.24). There is some evidence of a survival benefit for patients having chemotherapy (median survival 19.5 months with chemotherapy vs 13.5 months without, p=0.003). The effect is reduced when taking into account whether patients received radiotherapy (p=0.01), indicating that radiotherapy may reduce the overall benefit of the chemotherapy.

The Data Monitoring Committee (July 1999) recommended closing recruitment to the radiotherapy question. The trial continues to randomise between chemotherapy and observation to provide a reliable estimate of the difference between chemotherapy and surgery alone. The trial will roll into ESPAC-3, due to open June 2000 randomising between: (i) Surgery alone, (ii) 5 FU/FA, (iii) Gemcitabine.

### CT2 MEDICAL RESEARCH COUNCIL (MRC) RANDOMISED PHASE III TRIAL OF SURGERY WITH OR WITHOUT PRE-OPERATIVE CHEMOTHERAPY IN RESECTABLE CANCER OF THE OESOPHAGUS

PI Clark,* On behalf of the MRC Upper GI Tract Cancer Group, MRC Clinical Trials Unit, 222 Euston Road, London NW1 2DA, UK

**Background** The outlook for patients with oesophageal cancer undergoing surgical resection with curative intent remains poor, with approximately 20% alive at 2 years. There is increasing interest in the role of pre-operative chemotherapy.

**Methods** We conducted a randomised controlled trial (OE02) to assess the effects of pre-operative chemotherapy on survival and physical wellbeing. We planned to randomise 800 patients; this would enable an increase in 2-year survival from 20% to 30% to be detected with 5% significance level and 90% power.

**Results** Between March 1992 and June 1998, 802 previously untreated patients with resectable oesophageal cancer of any cell type, and fit for resection and chemotherapy, were randomised to either two 4-day cycles, 3 weeks apart, of cisplatin 80 mg/m$^2$ by 4-hour infusion plus fluorouracil 1 g/m$^2$/day by infusion for 4 days, followed by surgical resection (CS group), or surgical resection alone (S group). In the CS and S groups respectively, median age was 63 and 62 years; 77% and 74% were male; 66% and 67% had adenocarcinoma, 64% and 63% had lower third tumours, and WHO performance status was similar. Resection was considered complete in 84% CS compared with 71% S patients; and 9% and 10% died within 30 days of resection. Post-operative complications were similar (CS 38%, S 41%) and physical activity, dysphagia, and general wellbeing improved similarly in the two groups following treatment. In intent-to-treat analyses, overall survival was better in the CS group (hazard ratio [HR] 0.77; 95% confidence interval [CI] 0.64–0.91; p = 0.002). Median survival was 17.4 months compared with 13.4 months (difference 4 months; 95% CI 1.3–7.6 months), and 2-year survival rates were 45% and 35% (difference 10%; 95% CI 3%–16%). Progression-free survival was superior in the CS group (HR 0.71; 95% CI 0.60–0.84; p < 0.001), and there was no evidence of a different treatment effect according to histology.

**Conclusion** In conclusion, in the treatment of patients with resectable oesophageal cancer, two cycles of pre-operative cisplatin and fluorouracil improved survival without incurring additional serious adverse events.

### CT3 QUASAR: A UKCCCR STUDY OF ADJUVANT CHEMOTHERAPY (CT) FOR COLORECTAL CANCER

DJ Kerr, R Gray, C McConkey, J Barnwell, N Williams for the QUASAR group, CRC Inst. for Cancer Studies, University of Birmingham, UK

QUASAR is a large simple pragmatic trial which aims to determine which colorectal cancer patients should receive adjuvant chemotherapy (CT) and which CT to use. Patients with a clear indication for CT, and without metastases or other evident residual disease, were randomised in a 2×2 design to receive 5-fluorouracil (370 mg/m$^2$) with either high (175 mg) or low dose (25 mg) L-folinic acid, and coupled with either levamisole or placebo. The CT could be given, by clinician's choice, either as six-5-day courses at monthly intervals or as thirty once weekly doses. Patients for whom there is substantial uncertainty whether or not they should receive CT are randomised equally between CT and observation only with CT considered on recurrence.

The trial opened in May 1994 and has randomised 6409 patients from 145 centres in the UK and elsewhere. The CT comparisons closed in October 1997 with 4927 and 4863 randomised into the folinic acid dose and levamisole comparisons, respectively. The uncertain indication randomisation continues with 2100 patients randomised into a target of 2500 patients. The randomised comparisons suggest that there are no survival benefits from high-dose FA (p=0.43) or Levamisole (p=0.06 in favour of placebo) (Lancet, in press); analysis based on 1576 deaths and 1776 recurrences. Comparisons of the weekly and monthly schedules are also reported here. Although this is a non-randomised comparison, the weekly and monthly groups are well balanced with respect to prognostic variables. The weekly regime is much less toxic and, apparently, about as effective as the monthly schedule. This suggests that toxicity of FU/FA chemotherapy could be reduced substantially by weekly scheduling without comprising efficacy. Alternatively, efficacy might be enhanced with equal toxicity by more dose intense weekly FU regimens. But, this non-randomised comparison needs confirming in randomised studies.

### CT4 MITOXANTRONE IS SUPERIOR TO DOXORUBICIN IN A MULTI-AGENT WEEKLY REGIMEN FOR PATIENTS WITH HIGH-GRADE LYMPHOMA OVER THE AGE OF 60 YEARS: RESULTS OF A BNLI RANDOMISED TRIAL OF PACEBO VS. PMITCEBO

D Cunningham, PN Mainwaring, W Gregory, P Hoskin, B Hancock, P Smith, G Vaughan Hudson, D Linch, The British National Lymphoma Investigation at The CRC and UCL Cancer Trials Office, London, UK

A prospective, multicentre, randomised trial was undertaken to compare the efficacy and toxicity of adriamycin with mitoxantrone within a six drug combination chemotherapy regimen for elderly patients (≥ 60 years) with high-grade non-Hodgkin's lymphoma (HGL) given for a minimum of eight weeks. The adriamycin containing regimen (PACEBO) consisted of prednisolone 50 mg p.o. days 1–14, adriamycin 35 mg/m$^2$ IV day 1, cyclophosphamide 300 mg/m$^2$ IV day 1, etoposide 150 mg/m$^2$ IV day 1, bleomycin 10 mg/m$^2$ IV day 8 and vincristine 1.4 mg/m$^2$ day 8. In the PMitCEBO arm the adriamycin was replaced by mitoxantrone 7 mg/m$^2$ IV.

516 patients were entered into the trial and 473 patients were eligible for analysis. The overall and complete response rates were 78% and 60% for patients receiving PMitCEBO and 69% and 52% for patients receiving PAdriaCEBO, (P = 0.05, P = 0.12, respectively). Overall survival was significantly better with PMitCEBO compared with PAdriaCEBO (P = 0.0067). There was a trend towards improved lymphoma-specific survival in the PMitCEBO arm (P = 0.06), however, relapse-free survival was not significantly different (P = 0.16). At 4 years, 28% PAdriaCEBO patients and 50% of PMitCEBO patients were alive (P = 0.0001). Ann Arbor stage III/IV, WHO performance status 2–4 and elevated LDH negatively influenced overall survival from diagnosis. Significantly more patients died receiving PAdriaCEBO than PMitCEBO (137 vs. 107, P = 0.04) with treatment-related deaths and toxicity not significantly different between the two arms.

In view of these excellent results with PMitCEBO in elderly patients, a further BNLI trial in this group of patients involves a 2×2 randomisation of PMitCEBO vs CHOP with a second randomization to G-CSF or not. 412 patients have been entered to date with a target accrual of 650 patients.

### CT5 PREOPERATIVE CONTINUOUS INFUSIONAL (EPIRUBICIN, CISPLATIN AND INFUSIONAL 5 FU) V. CONVENTIONAL AC CHEMOTHERAPY FOR EARLY BREAST CANCER: AS PHASE III RANDOMISED TRIAL

IE Smith, RP A'Hern, S Ebbs, A Howell, T Hickish, M O'Brien, A Robinson, C Wilson, Royal Marsden Hospital, London: Mayday Hospital, Croydon; Christie Hospital, Manchester; Royal Bournemouth Hospital; Kent Cancer Centre; Southend Hospital; Addenbrooke's Hospital, Cambridge; UK on behalf of TOPIC Trial Group

Continuous infusional (ci) ECisF (E 60 mg/m$^2$ iv bolus, Cis 60 mg/m$^2$ iv, both × 3 weekly (wk) × 6 courses, with ci 5 FU 200 mg/m$^2$ × 24 hourly × 18 wk by ambulatory pump) is highly active preoperatively with an overall response rate of 98% and complete remission 66% (J Clin Oncol 13: 424, 1995). We have therefore compared ci ECisF with conventional AC (Adriamycin 60 mg/m$^2$, Cyclophosphamide 600 mg/m$^2$ both iv × 3 wk × 6 courses) in a phase III multicentre randomised trial (TOPIC). 426 pts with needle biopsy proven invasive operable ≥3 cm breast cancer were randomised to receive either ci ECisF (211 pts) or AC (215 pts), followed by appropriate local surgery ± radiotherapy and tamoxifen 20 mg daily × 5 years. Patient characteristics for ci ECisF v. AC respectively were as follows: median age 46 (range 22–68) v. 47 (range 25–66) yrs, premenopausal 64% v. 66%, median tumour diameter 5 (range 3–11) v. 5 (3–15) cm. Results at median follow-up of 30 months for ECisF v. AC respectively were as follows:

| | ci ECisF | AC | |
|---|---|---|---|
| Complete Remission | 34% | 31% | p=0.53 |
| Overall Response | 77% | 75% | p=0.56 |
| 3-yr Mastectomy Rate | 37% | 43% | p=0.13 |
| 3-yr Relapse Free Survival | 77% | 66% | HR 0.77 (0.52–1.14) p=0.19 |
| 3-yr Overall Survival | 90% | 80% | HR 0.56 (0.32–0.97) p=0.04 |

Grade 3/4 toxicity was low in both arms but significantly worse for ECisF for nausea and vomiting (21% v. 10%), diarrhoea (7% v. 2%), thrombosis (17% v. 2%), palmar/plantar erythema (14% v. 1%), and significantly better for alopecia (with scalp cooling) (51% v. 74%). This interim analysis shows a significant survival benefit for infusional 5 FU-containing chemotherapy over conventional AC, despite no significant difference in clinical complete remission or overall response rates. Non-clinical parameters (pathological or biological, which have run in parallel) may predict better for survival.

### CT6 ROLE OF RADIOTHERAPY AND DURATION OF TAMOXIFEN IN EARLY BREAST CANCER (STAGE I): WEST MIDLANDS BREAST GROUP PROSPECTIVE RANDOMISED COLLABORATIVE STUDY (BR3002)

D Spooner, JA Dunn, JM Morrison, GD Oates, DR Ellis, JR Lee. A Aukland, RJ Grieve, RJ Blunt, HM Bishop, L Dodson, On behalf of the West Midlands Breast Group. CRC Trials Unit, Institute of Cancer Studies, University of Birmingham B15 2TA, UK

Between August 1985 and December 1992, 707 patients with early breast cancer (less than 4 cm diameter with clinically negative axillae) were treated with wide local excision only and then randomised to receive immediate post-operative radiotherapy to the breast and axilla or observation only. All patients were further randomised to stop Tamoxifen after 2 years or continue.

All patients now have a minimum of 5-year follow-up, median 8 years [IQR 6.4–9.7] years. The radiotherapy results show an expected excess of local relapses in the no radiotherapy arm ($\chi^2$=22.9, P<0.0001) which has a significant impact on breast cancer specific survival (P=0.04) and a trend on overall survival (P=0.06). Looking at the duration of Tamoxifen, there is no significant excess of recurrences (P=0.24) or deaths (P=0.11) in either arm but again a trend in favour of continuous Tamoxifen. These trends hold true when stratifying by prognostic factors. The compliance with Tamoxifen allocation will also be presented.

This is a mature, important study looking at the impact of conservation treatment. These results looking at the role of radiotherapy and duration of Tamoxifen enforce those shown in the Oxford Overviews.

### CT7 CHEMOTHERAPY IN HIGH-GRADE GLIOMA: A META-ANALYSIS USING INDIVIDUAL PATIENT DATA FROM RANDOMISED CLINICAL TRIALS (RCTS)

LA Stewart, S Burdett[1], RL Souhami[2], on behalf of the Glioma Meta-analysis Trialists Group, [1]MRC Clinical Trials Unit, London, UK, [2]University College London Medical School, London, UK

A prospectively defined systematic meta-analysis assessed the role of chemotherapy in the treatment of adult patients with high-grade glioma. Individual patient data were obtained from 10 RCTs comparing radiotherapy alone with radiotherapy plus chemotherapy, mostly nitrosoureas either alone or in combination. The meta-analysis included 2368 patients and 2106 deaths. Data were combined using the stratified (by trial) log rank test to calculate pooled hazard ratios (HRs). Absolute differences were calculated from each HR and the corresponding control group event rate at 2 years.

| Endpoint | HR (95% CI) | Absolute Difference At 2 years | p-value |
|---|---|---|---|
| Survival | 0.84 (0.77–0.92) | 5% (from 15 to 20%) | 0.0001 |
| Recurrence-free survival | 0.80 (0.72–0.90) | 6% (from 10 to 16%) | 0.00008 |

The results show a significant benefit of chemotherapy with a 16% relative reduction in the risk of death. This is equivalent to an absolute improvement of 5% at 2 years (95% confidence interval 3 to 9%) increasing the survival rate from 15% to 20%. There was no evidence that the effect of chemotherapy was different in any group of patients defined by age, sex, histology, performance status or extent of resection. This small but clear improvement in survival from chemotherapy encourages further study of systemic treatment of these tumours.

### CT8 AUDIT OF CLINICAL TRIAL ELIGIBILITY AND RECRUITMENT RATES IN A SINGLE SPECIALITY CANCER CENTRE

TS Maughan*[1,2], L Branston[2], LA Batt[1] and SH Shankland[1,2], Clinical Trials Unit, Velindre NHS Trust, Whitchurch, Cardiff CF14 2TL, [2]Wales Cancer Trials Network, Velindre NHS Trust, Whitchurch, Cardiff CF14 2TL, UK

This abstract reports a retrospective audit of cancer trial activity in a single speciality cancer centre in South East Wales. The audit included all referrals to the hospital in October 1998 and March 1999. The medical summary sheet for each patient referred was reviewed by specialist oncology research nurses with a view to determining whether the patient was potentially eligible for any of the 40+ open trials running in the hospital at the time. Each summary sheet was coded according to the scheme below and difficult cases were reviewed in audit meetings. The results are summarised in the table below.

| | Oct 98 | Mar 99 | TOTAL |
|---|---|---|---|
| Assessable records | 343 | 364 | 707 |
| Not eligible/no trial available | 238 | 289 | 527 (74.5% of referrals) |
| Potentially eligible | 83 | 80 | 163 (23% of referrals) |
| Eligible but not approached | 31 | 41 | 72 (44% of eligible) |
| Eligible & declined | 33 | 24 | 57 (35% of eligible) |
| Eligible and in trial | 19 | 15 | 34 (21% of eligible) 4.8% of all referrals |

Over 70% of patients were ineligible for trial entry from the outset because of poor performance status, stage of disease or no trial being available for certain cancers. However, the audit helped highlight areas where interventions could be targeted to increase recruitment of the remaining potentially eligible patients. These could include educating medical personnel about the trial portfolio, giving training in informed consent where refusal rates are high, screening notes and identifying possible trials before the consultant sees the patient and support to satellite clinics where there were patients who were not approached about trials.

A study by Spiro et al.[1] at two centres with a major interest in lung cancer reported that 11.7% of patients referred were randomised into the Big Lung Trial. Whilst the authors judged this to be disappointing, it compares favourably with the 4.8% in this audit. It would be useful to repeat audits of this kind in other settings to establish achievable ranges for trial recruitment in UK cancer hospitals.

1 Spiro SG, Gower NH, Evans MT, Facchini FM, Rudd RM. Recruitment of Patients with Lung cancer into a Randomised Clinical Trial: Experience at 2 Centres. *Thorax* (in press)

